# Anti-inflammatory activity of *Acanthospermum australe*: Insights from network pharmacology, chemical analysis, and *in vitro* assays

**DOI:** 10.1371/journal.pone.0337712

**Published:** 2025-11-26

**Authors:** Andrea Salinas, Christa Burgos, Aaron Rodríguez-Ramos, Alberto Burgos-Edwards, Nelson Alvarenga, Pablo H. Sotelo, Patricia Langjahr

**Affiliations:** 1 Department of Biotechnology, Facultad de Ciencias Químicas, Universidad Nacional de Asunción, Paraguay; 2 Department of Phytochemistry, Facultad de Ciencias Químicas, Universidad Nacional de Asunción, Paraguay; Airlangga University Faculty of Medicine: Universitas Airlangga Fakultas Kedokteran, INDONESIA

## Abstract

Inflammation plays a crucial role in homeostasis and defense responses; however, exaggerated and chronic inflammation contribute to the development and worsening of various diseases. *Acanthospermum australe* (Loefl.) Kuntze (*A. australe*) is a medicinal plant traditionally used to alleviate inflammation. However, the anti-inflammatory activity of this plant has not yet been explored. This study aimed to evaluate the immunomodulatory activity of this species using network pharmacology, UPLC-ESI-MS/MS analysis, and *in vitro* assays. Network pharmacology analysis revealed the involvement of immune system processes, and among the main targets of *A. australe* related to inflammation were innate immune responses, toll-like receptors (TLRs), and T cell receptor signaling pathways. A methanolic extract was prepared and analyzed using UPLC-ESI-MS/MS, and 15 compounds were detected. Additionally, the potential targets of *A. australe* predicted by network pharmacology analysis were validated *in vitro* using monocytic THP-1 cells and splenocytes. The RT-qPCR analysis indicated that *A. australe* significantly inhibited the production of pro-inflammatory cytokines IL-1β, IL-6, and TNF-α, as well as chemokine CCL-2, in lipopolysaccharide (LPS)-stimulated cells. Finally, the extract significantly decreased concanavalin A (ConA)-induced T cell proliferation. Overall, our study provides evidence for the anti-inflammatory effects of this species and highlights its mechanisms of action.

## Introduction

Inflammation is an essential defense response against infections, tissue damage, and other aggression. Inflammatory processes involves components of innate and adaptive immunity, which cause the release of inflammatory mediators such as interleukin (IL)-1β, IL-6, and tumor necrosis factor-alpha (TNF-α) [1]. However, excessive inflammation can lead to chronic diseases, including autoimmune diseases, atherosclerosis, and cancer. Indeed, inflammation has been shown to control the onset and progression of many complex diseases. Notably, a large proportion of deaths worldwide can be attributed to inflammation-related diseases, including diabetes, cancer, stroke, chronic kidney disease, and autoimmune and neurodegenerative diseases, among others [2,3].

Nonsteroidal anti-inflammatory drugs (NSAIDs) and corticosteroids are widely used to treat inflammatory conditions. However, the prolonged use of NSAIDs and corticoids is associated with side effects and these drugs have low efficacy in some patients [4–6]. These issues make the treatment of inflammatory and autoimmune disorders one of the most challenging areas of medicine, and efforts are ongoing to identify alternative therapeutic options that are both safer and more efficient [7]. In this context, medicinal plants have emerged as invaluable reservoirs for the innovation and formulation of novel therapeutic agents. Owing to the extensive historical use of such medicinal plants in the management of inflammatory diseases, using them as sources of pharmacotherapeutic agents offers several advantages, including potentially high safety [8].

*Acanthospermum australe* (Loefl.) Kuntze (*A. australe*), belonging to the family *Asteraceae* and popularly known as “tape kue”, is a medicinal plant native to the tropics and subtropics of Central and South America, although it is also common throughout North America and Asia. This species is used in traditional medicine to treat inflammation (gout, arthritis, eczema, and skin affections in general) [9,10].

The chemical composition of *A. australe*, which includes tannins, flavonoids, saponins, and phenolic compounds, has been previously reported [11–13]. Studies have also reported its antiviral [13,14], antibacterial [11], antifungal [15,16], antiparasitic [17], and antitumour [18] properties. Moreover, the antioxidant activity of this plant has been identified using *in vitro* assays [19]. However, the effects of *A. australe* on inflammation have not been explored. As such, it is important to study these effects in order to validate its popular use and to better understand its anti-inflammatory mechanisms.

Although studying these anti-inflammatory effects is crucial, the intricate complexity of the chemical composition of plants, which often comprises a diverse array of molecules, poses a significant challenge in the development of new therapeutic agents. This chemical complexity arises from the inherent diversity of the phytochemicals present in plants, which encompass a broad spectrum of compounds. In this context, a network pharmacology strategy provides a useful solution to this problem; indeed this bioinformatic approach is a potent research strategy for exploring the therapeutic effects and targets of medicinal herbs [20].

In this study, we aimed to explore, for the first time, the anti-inflammatory activity of *A. australe* using network pharmacology tools to predict possible molecular targets related to inflammation. This network pharmacology was also integrated with UPLC-ESI-MS/MS analysis and *in vitro* assays, in order to verify the anti-inflammatory activity of *A. australe* and explore the mechanisms of its immunomodulatory activity.

## Materials and methods

### Plant material extraction

*A. australe* was collected from the Central and Cordillera Departments of Paraguay, identified following the procedure described by Degen de Arrúa et al. [10], and deposited in the Herbarium-FCQ at the Department of Botany, Facultad de Ciencias Químicas, Universidad Nacional de Asunción (voucher specimen number 4061). For all of the experimental assays, a methanolic extract of the aerial parts of *A. australe* was obtained as previously described [21]. Briefly, the plant material was air-dried and ground into small pieces. Subsequently, the extract was prepared by ultrasound-assisted maceration, during which the material was suspended in HPLC-grade methanol (Sigma, USA) at a ratio of 1.5 L per 200 g and sonicated for 30 min; this process was repeated three times. The mixture was left to stand overnight and then filtered through a qualitative filter paper. The extraction procedure was repeated over 2 days, after which the filtrates were combined, and the solvent was removed. A rotary evaporator (RVO 400 SD, Boeco, Germany) was used to obtain the crude extract. The extract was weighed, the yield was calculated (14.40%), and then, the extract was diluted in dimethyl sulfoxide (DMSO).

### Prediction of compound-related targets

A search for compounds in the species was conducted using the Lotus Natural Products website (https://lotus.naturalproducts.net/), SDF files of the 41 identified compounds were obtained. The SwissADME tool (http://www.swissadme.ch/) was employed to evaluate the absorption, distribution, metabolism, and excretion (ADME) properties of the constituents. Compounds were selected if they met at least three of the five criteria associated with Lipinski’s rule [22].

The prediction of target molecules was performed using the Swiss TargetPrediction server. Compounds that did not meet the criteria due to the number of atoms per compound in the algorithm were excluded, resulting in a probability value of less than 0.1. The 31 compounds fulfilling the described criteria were compared with a homemade database of cellular proteins related to inflammation. To create a comprehensive database of genes linked to inflammation, an extensive search was performed using the keyword “inflammation” in both the Human Gene Database (GeneCards) and Online Mendelian Inheritance in Man (OMIM) database. The results from these databases were then merged and standardized using UniProt codes for each identified gene. Finally, the overlapping genes between the predicted targets of the compounds and those related to inflammation were identified using a Venn Diagram tool (https://www.bioinformatics.com.cn/).

### Network construction and analysis

A protein-protein interaction network of *A. australe* targets was constructed using Cytoscape 3.9.1. For this, the STRING tool with “homo sapiens” and a confidence score higher than 0.4 was used. The network obtained by STRING was analyzed using the Molecular Complex Detection (MCODE) tool to obtain highly interconnected protein clusters.

### Pathway and functional enrichment analysis

Functional enrichment analysis of the protein-protein interaction networks was conducted using the genome as a reference network and employing default parameters for flat node design. This analysis aimed to identify the biological processes and molecular pathways associated with the target proteins within each cluster in the KEGG database. From this analysis, 20 pathways with the lowest p-values were selected, and a bubble plot was generated using the ggplot2 package in R Studio.

### HPLC-DAD analysis

To analyze the extract, high-performance liquid chromatography with a diode array detector (HPLC-DAD) was conducted using a Shimadzu chromatographer (Shimadzu Corporation, Japan), which was equipped with an LC-20AT pump, an SPD-M20A UV diode array detector, and a CTO-20 AC column oven. The system was operated using LabSolution software. An Inertsil ODS-4 column (5 μm, 250 × 4.6 mm, GL Sciences Inc., Japan) was employed and maintained at a temperature of 40 °C. During the procedure, 5 mg of the extract was dissolved in 1 mL of a methanol:water (MeOH:H_2_O, 1:1, v/v) mixture, filtered through a 0.22 µm nylon filter (Merck, Germany), and subsequently injected into the chromatographic system (injection volume = 20 µL). The analysis was performed using a linear gradient solvent system at a flow rate of 0.5 mL/min. The solvent system comprised water with 0.1% formic acid (A) and methanol with 0.1% formic acid (B). The analytes were eluted according to the following gradient: 0–5 min at 80% A, 5–23 min at a linear gradient from 80% to 60% A, 23–55 min at a linear gradient from 60% to 20% A, and 55–60 min at a linear gradient from 20% to 0% A. Finally, the initial conditions were held constant for 5 min. Spectrophotometry was performed at 280 nm.

### UPLC-ESI-MS/MS analysis

Ultra-performance liquid chromatography–electrospray tandem mass spectrometry (UPLC-ESI-MS/MS) was conducted using a Waters Acquity UPLC system (Milford, USA) coupled with a Xevo TQD QqQ-MS mass detector. Chromatographic separations were performed on a Phenomenex KINETEX core-shell EVO-C18 (2.1 mm × 100 mm, 1.7 μm) at a flow rate of 0.3 mL/min. The column temperature was maintained at 40 °C. The mobile phase consisted of methanol (A) and water (B), both of which contained 0.1% formic acid and 10 mM ammonium formate. The gradient was programmed as follows: 0–0.7 min, 80% A; 0.7–3.2 min, 80% to 60% A; 3.2–7.6 min, 60% to 20% A; 7.6–8.3 min, 20% to 0% A; 8.3–10.4 min, 0% to 80% A; and 10.4–12 min, 80% A. Mass spectra between m/z 80 and 800 were identified using the ESI negative ion mode. Argon and nitrogen were used as the collision and nebulizer gases, respectively. The parameters for the analysis were as follows: electrospray capillary voltage of 2.5 kV, source temperature of 150 °C; desolvation temperature of 350 °C; cone voltage of 30 V, cone gas flow of 80 L/h, and desolvation gas flow of 900 L/h. The collision energy (CE) was optimized as follows: 20 V for hydroxycinnamic esters, 25 V for flavonol derivatives, and 30 V for aglycones. The data were processed using Waters Masslynx V4.1 software.

### Cell culture

The human monocyte cell line THP-1 and murine splenocytes were used as cell models. The THP-1 cells were maintained in RPMI 1640 medium supplemented with 10% fetal bovine serum and 1x penicillin and streptomycin (Sigma, USA) at 37 °C and 5% CO_2_. Splenocytes were isolated from the spleens of three 7-week-old female Swiss albino mice euthanized by cervical dislocation under sodium pentobarbital anesthesia. For the isolation procedure, the spleen was extracted, macerated, and passed through a 70 µm cell strainer (Falcon®, USA). The cells were then centrifuged, the pellets were treated with ACK lysis buffer, and two washes were performed with 1x PBS. Following this, the cells were resuspended in supplemented RPMI 1640 medium and passed through a 70 µm cell strainer. Finally, cell viability was assessed using trypan blue staining and counting the cells with a Neubauer chamber.

### Cytotoxicity assay

The cytotoxicity of the methanolic extract on THP-1 cells and murine splenocytes was evaluated. In this assay, THP-1 cells (1 × 10^6^ cells/mL) were incubated with different concentrations of *the A. australe* extract for 20 h at 37 °C and 5% CO_2_. Subsequently, an MTT reagent (3-(4,5-dimethylthiazol-2-yl)-2,5-diphenyltetrazolium bromide) was added for 3 h at 37 °C and 5%. To evaluate the cytotoxic effects of the methanolic extract on murine splenocytes, the cells (5 × 10^6^ cells/mL) were incubated with different concentrations of the extract at 37 °C and 5% CO_2_. After 48 h of incubation, resazurin was added, and the cells were incubated for 24 h. Cells that were not treated or were treated with the vehicle (DMSO) were used as controls. The absorbance of each plate was measured at 570 nm and 630 nm, and cell viability was determined as a percentage of live cells.

### Quantitative reverse transcription polymerase chain reaction (RT-qPCR)

The gene expression levels of the inflammatory mediators were measured using qPCR. THP-1 cells were cultured at 1 × 10^6^ cells/mL and incubated with 50 μg/mL *A. australe* extract and 1 μg/mL bacterial lipopolysaccharide (LPS) (*E. coli* O111:B4, Sigma Aldrich, USA) for 4 h at 37°C and 5% CO_2_. Cells that were treated with LPS alone were used as controls. Total RNA was extracted using the TRIzol reagent (Thermo Fisher Scientific, USA), according to the manufacturer’s instructions. RT-qPCR was performed using the M-MLV Reverse Transcriptase Kit (Promega) and SsoAdvanced Universal SYBR Green Supermix (Bio-Rad). The specific primers for each gene were as follows: TNF-α F: TCCTTCAGACACCCTCAACC, R: AGGCCCCAGTTTGAATTCTT; IL-1β F: TGATGGCTTATTACAGTGGCAATG, R: GTAGTGGTGGTCGGAGATTCG [23]; IL-6 F: TCTCCACAAGCGCCTTCG, R: CTCAGGGCTGAGATGCCG [24]; and MCP-1 F: GATCTCAGTGCAGAGGCTCG, R: TGCTTGTCCAGGTGGTCCAT [25]. RPL13A (F: CATCGTGGCTAAACAGGTACTG; R: CGCACGACCTTGAGGGCAGC) [26] was used as the normalizer. The relative mRNA expression was determined using the 2^-ΔΔCT^ method.

### Splenocyte proliferation assay

For the splenocyte proliferation assay, murine splenocytes were cultured at 5 × 10^6^ cells/mL in 96-well plates and treated with different extract concentrations. After 48 h of incubation, the cells were treated with resazurin, and the absorbance was measured after 24 h at 570 and 630 nm. Concanavalin A was used as the proliferation control. To evaluate cell proliferation, the proliferation index (PI) was calculated using the following formula:


$$PI=(\varepsilon OX)\lambda 2A\lambda 1-(\varepsilon OX)\lambda 1A\lambda 2\;treated\;cells\;/\;(\varepsilon OX)\lambda 2A^{\circ }\lambda 1-(\varepsilon OX)\lambda 1A^{\circ }\lambda 2\;control\;(ConA)\mathrm{\backslash ]}$$

where PI is the proliferation index, εOX is the molar extinction coefficient of oxidized resazurin, A is the absorbance of the treated cells, A°is the absorbance of the control cells, and λ1 = 570 nm and λ2 = 600 nm.

### Ethical considerations

The Ethics Committee of the FCQ, Universidad Nacional de Asunción approved the experimental study (Approval Number 798/21).

### Statistical analysis

The data were analyzed using the analysis of variance (ANOVA) using the GraphPad 8 software. All experiments were repeated at least three times (n = 3). The results are expressed as arithmetic means ± standard deviations. Statistical significance was set at P < 0.05.

## Results

Initially, 41 compounds present in *A. australe* were identified through the Lotus database search. ADME analysis was subsequently performed, and only rutin and 3-rutinosyl quercetin showed three violations of Lipinski’s rule (Table 1). Therefore, these compounds were excluded from further analysis.

**Table 1:** Predicted ADME properties of the *A. australe* compounds.

Compound	Lotus ID	Number of violations of Lipinski rules
5,7-dihydroxy-2-(4-hydroxyphenyl)-3,6-dimethoxychromen-4-one	LTS0121397	0
(3as,4s,5s,11ar)-6-formyl-10-methyl-3-methylidene-4-[(2-methylpropanoyl)oxy]-2-oxo-3ah,4h,5h,8h,9h,11ah-cyclodeca[b]furan-5-yl 2-hydroxy-2-methylpropanoate	LTS0273651	0
N-[(3ar,4s,5s,11ar)-6-formyl-4-hydroxy-10-methyl-3-methylidene-2-oxo-3ah,4h,5h,8h,9h,11ah-cyclodeca[b]furan-5-yl]-2-methylpropanimidic acid	LTS0196950	0
6-formyl-10-methyl-3-methylidene-4-[(2-methylpropanoyl)oxy]-2-oxo-3ah,4h,5h,8h,9h,11ah-cyclodeca[b]furan-5-yl 2-hydroxy-2-methylpropanoate	LTS0012956	0
(2e)-5-[(2s,3r)-3-hydroxy-3-methyl-6-(4-methylpent-3-en-1-yl)-7-oxo-2,4-dihydrooxepin-2-yl]-3-methylpent-2-en-1-yl acetate	LTS0226006	0
(2e,6r)-6-hydroxy-3-methyl-6-[(2s)-2-methyl-6-(4-methylpent-3-en-1-yl)-7-oxo-3,4-dihydrooxepin-2-yl]hex-2-en-1-yl acetate	LTS0079843	0
Penduletin	LTS0080627	0
(1as,4as,7s,7ar,7bs)-1,1,7-trimethyl-4-methylidene-octahydrocyclopropa[e]azulen-7-ol	LTS0073517	0
Phytol	LTS0031808	1
Rutin	LTS0042292	3
Hyperoside	LTS0089156	2
(3ar,4s,5s,11as)-6-formyl-10-methyl-3-methylidene-4-[(2-methylpropanoyl)oxy]-2-oxo-3ah,4h,5h,8h,9h,11ah-cyclodeca[b]furan-5-yl 2-hydroxy-2-methylpropanoate	LTS0072121	0
(3as,4s,5s,11ar)-6-formyl-5-methoxy-10-methyl-3-methylidene-2-oxo-3ah,4h,5h,8h,9h,11ah-cyclodeca[b]furan-4-yl 2-methylpropanoate	LTS0062693	0
(3ar,11ar)-6-formyl-10-methyl-3-methylidene-2-oxo-3ah,4h,9h,11ah-cyclodeca[b]furan-4-yl 2-methylpropanoate	LTS0180396	0
6-(hydroxymethyl)-10-methyl-3-methylidene-4-[(2-methylpropanoyl)oxy]-2-oxo-3ah,4h,5h,8h,9h,11ah-cyclodeca[b]furan-5-yl 2-hydroxy-2-methylpropanoate	LTS0184872	0
2-(3,4-dihydroxyphenyl)-5,7-dihydroxy-3-{[3,4,5-trihydroxy-6-(hydroxymethyl)oxan-2-yl]oxy}chromen-4-one	LTS0195312	2
(3ar,4r,11ar)-6-formyl-10-methyl-3-methylidene-2-oxo-3ah,4h,9h,11ah-cyclodeca[b]furan-4-yl 2-methylpropanoate	LTS0090650	0
6-formyl-5-hydroxy-10-methyl-3-methylidene-2-oxo-3ah,4h,5h,8h,9h,11ah-cyclodeca[b]furan-4-yl 2-methylpropanoate	LTS0177245	0
(3as,4r,11ar)-6-formyl-10-methyl-3-methylidene-2-oxo-3ah,4h,9h,11ah-cyclodeca[b]furan-4-yl 2-methylpropanoate	LTS0190404	0
(3as,4s,5s,11ar)-6-formyl-10-methyl-3-methylidene-4-[(2-methylpropanoyl)oxy]-2-oxo-3ah,4h,5h,8h,9h,11ah-cyclodeca[b]furan-5-yl 2-hydroxy-2-methylpropanoate	LTS0141233	0
3,4-dihydroxycinnamic acid	LTS0128050	0
Germacrene c	LTS0161450	0
6-formyl-10-methyl-3-methylidene-2-oxo-3ah,4h,9h,11ah-cyclodeca[b]furan-4-yl 2-methylpropanoate	LTS0232029	0
6-hydroxy-3-methyl-6-[2-methyl-6-(4-methylpent-3-en-1-yl)-7-oxo-3,4-dihydrooxepin-2-yl]hex-2-en-1-yl acetate	LTS0100695	0
10-methyl-4,5-bis[(2-methylbut-2-enoyl)oxy]-3-methylidene-2-oxo-3ah,4h,5h,8h,9h,11ah-cyclodeca[b]furan-6-carboxylic acid	LTS0262015	0
6-formyl-10-(hydroxymethyl)-4-[(2-methylbut-2-enoyl)oxy]-3-methylidene-2-oxo-3ah,4h,5h,8h,9h,11ah-cyclodeca[b]furan-5-yl 2-methylbut-2-enoate	LTS0180646	0
(3as,4s,5s,11ar)-10-methyl-4,5-bis({[(2z)-2-methylbut-2-enoyl]oxy})-3-methylidene-2-oxo-3ah,4h,5h,8h,9h,11ah-cyclodeca[b]furan-6-carboxylic acid	LTS0180048	0
5-[3-hydroxy-3-methyl-6-(4-methylpent-3-en-1-yl)-7-oxo-2,4-dihydrooxepin-2-yl]-3-methylpent-2-en-1-yl acetate	LTS0268893	0
Trifolin	LTS0237581	2
Thymol	LTS0168527	0
(3as,4s,5s,11ar)-6-formyl-10-(hydroxymethyl)-4-{[(2z)-2-methylbut-2-enoyl]oxy}-3-methylidene-2-oxo-3ah,4h,5h,8h,9h,11ah-cyclodeca[b]furan-5-yl (2z)-2-methylbut-2-enoate	LTS0225528	0
(7r)-7-[(1r,4e)-1,6-dihydroxy-4-methylhex-4-en-1-yl]-7-methyl-3-(4-methylpent-3-en-1-yl)-5,6-dihydrooxepin-2-one	LTS0018008	0
Caffeic acid	LTS0027481	0
(7s)-7-[(1r,4z)-1,6-dihydroxy-4-methylhex-4-en-1-yl]-7-methyl-3-(4-methylpent-3-en-1-yl)-5,6-dihydrooxepin-2-one	LTS0158066	0
(-)-germacrene a	LTS0022487	1
Chrysosplenol d	LTS0188255	0
Axillarin	LTS0067813	0
Quercetin	LTS0004651	0
(1s,2e,10r)-3,7,11,11-tetramethylbicyclo[8.1.0]undeca-2,6-diene	LTS0032090	1
3-rutinosyl quercetin	LTS0032845	3
7-(1,6-dihydroxy-4-methylhex-4-en-1-yl)-7-methyl-3-(4-methylpent-3-en-1-yl)-5,6-dihydrooxepin-2-one	LTS0228581	0

Subsequently, the potential cellular targets of the *A. australe* compounds were predicted using the Swiss TargetPrediction server and the targets related to inflammation were identified (S1 Table). The STRING database was employed to construct a comprehensive protein-protein interaction (PPI) network among the previously identified targets. The resulting PPI network comprised 666 nodes and 12,696 interaction edges, with an average node degree of 38.13. The P-value obtained for PPI clustering was 1.0e-16, highlighting the biological significance and integrity of the network. The functional enrichment analysis revealed the involvement of the targets in diverse biological processes, particularly in the immune system (S2 Table).

**Fig 1:**
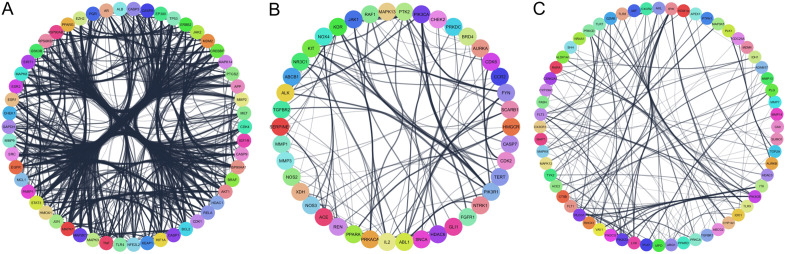
Protein–protein interaction (PPI) network of the identified targets of the three (A-C) prominent clusters.

**Fig 2:**
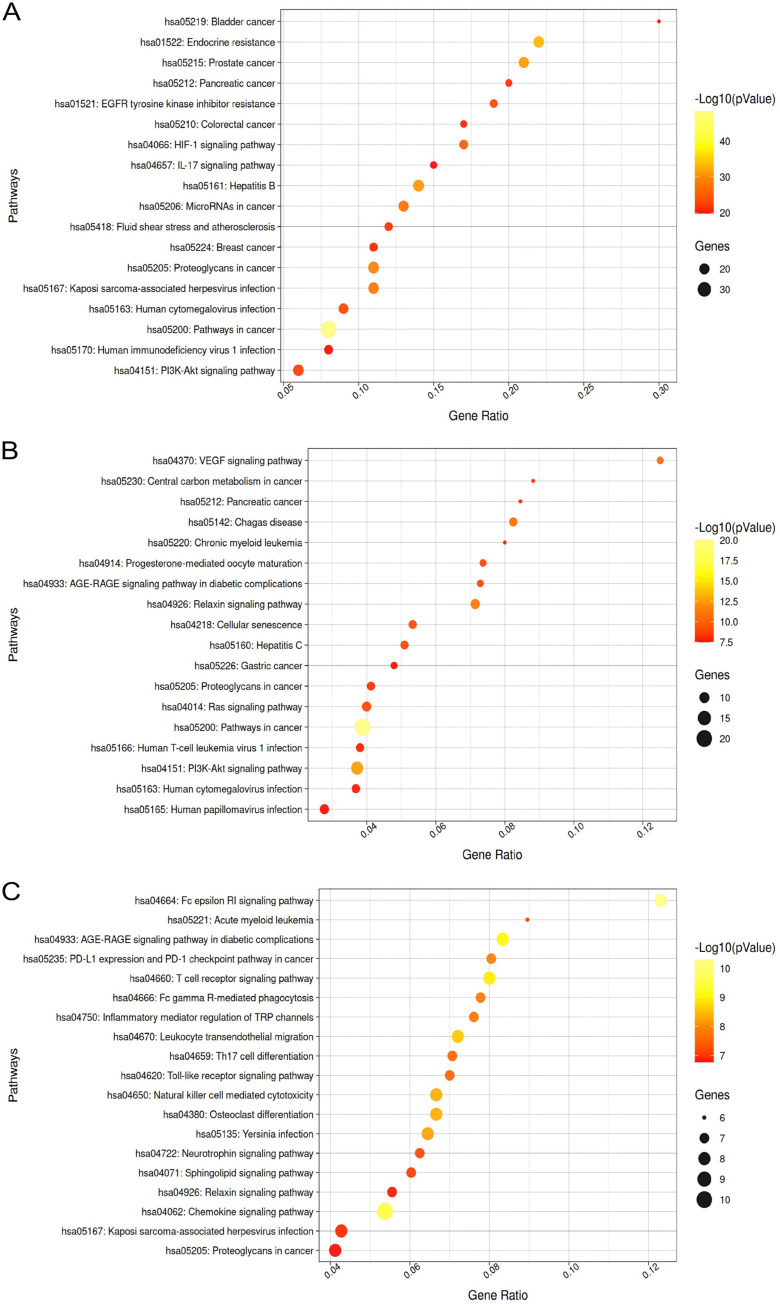
Bubble plot of the KEGG pathway enrichment analysis of clusters 1 (A), 2 (B), and 3 (C).

Given the complexity of the PPI network, an analysis was conducted using MCODE to identify highly interconnected protein clusters. This analysis revealed three prominent clusters (Fig 1). The KEGG functional enrichment analysis of these clusters revealed the involvement of the immune system processes, including innate immune responses (Fig 2). Notably, in cluster 3, the analysis predicted the involvement of relevant pathways such as T-lymphocyte receptor activation, cytokine signaling, and the toll-like receptor (TLR) pathway (Fig 2C).

**Fig 3:**
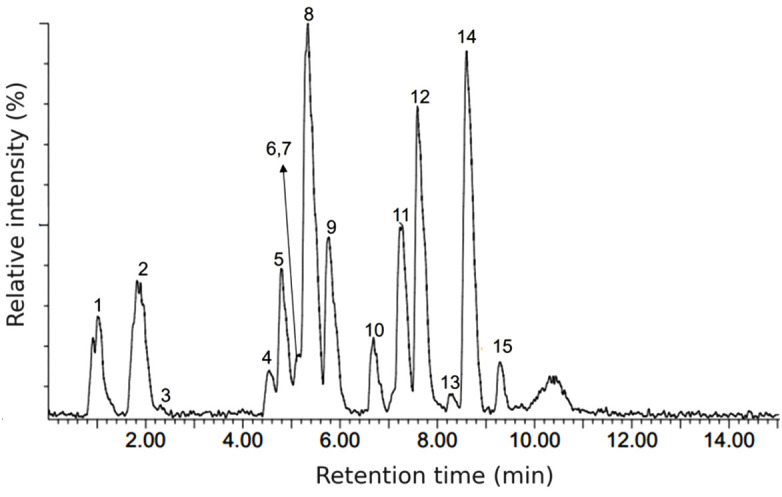
BPI chromatogram of the MeOH extract from *Acanthospermum australe* in negative ion mode.

Although network pharmacology is an efficient method for predicting different targets of *A. australe* compounds, these conclusions had to be experimentally confirmed by examining the anti-inflammatory activity of *A. australe*, which had previously not been explored. The UPLC-ESI-MS/MS analysis of the prepared *A. australe* extract detected 15 compounds (Fig 3). A list of these compounds is provided in S3 Table, and the S4 and S5 Figs include the spectral data and extracted ion chromatograms, respectively. Overall, 12 of the compounds were tentatively identified based on their mass spectra, ultraviolet absorption, and literature reports, when available. These compounds included six hydroxycinnamic acids, three flavanol derivatives, and three methylated flavones. The major peak (peak 8) in the chromatogram was assigned to a dicaffeoylquinic acid (Fig 3). The identified compounds were part of those used for the network pharmacology analysis.

**Fig 4:**
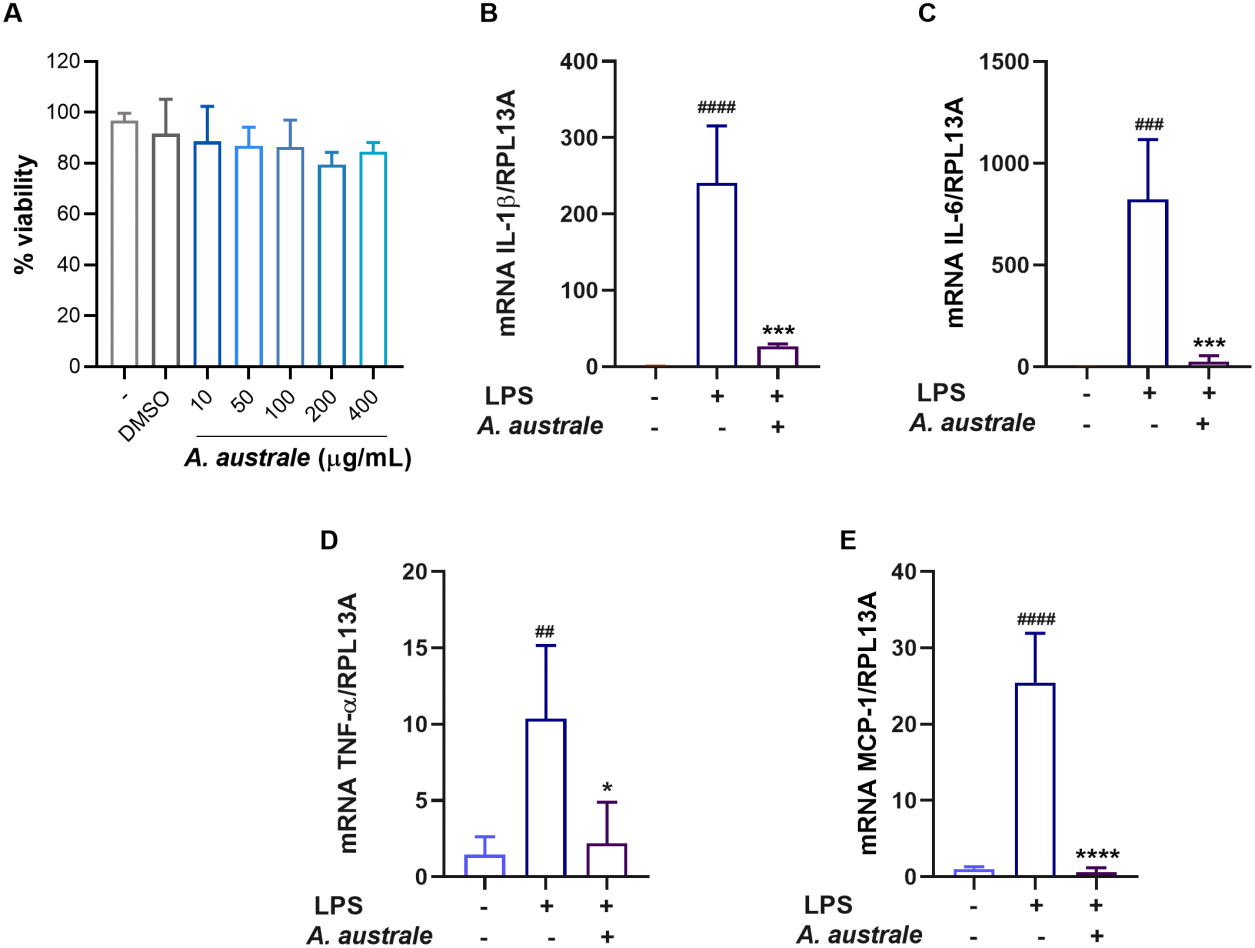
Effects of *A. australe* on cytokine expression in LPS-stimulated THP-1 cells. **A)** Effect of *A. australe* extract on cell viability. The cells were treated with different concentrations of the extract for 20 **h.** B-E) Effect of *A. australe* on IL-1β, IL-6, TNF-α, and MCP-1 mRNA levels. ANOVA (n = 4), ## P < 0.01, ### P < 0.001, #### P < 0.0001 vs. untreated cells. *P < 0.05, ***p < 0.001, ****p < 0.0001 vs. LPS-treated cells.

Since the TLR signaling pathway was identified as a potential target of *A. australe*, the ability of the extract to inhibit the production of pro-inflammatory mediators following the activation of the TLR4 signaling by LPS in monocytes was evaluated. As shown in Fig 4A no decrease in cell viability was observed in response to *A. australe* treatment (10, 50, 100, 200, and 400 µg/mL). Subsequently, we analyzed the effect of the extract on cytokine and chemokine production in LPS-stimulated THP-1 cells. The extract significantly reduced the mRNA levels of the cytokines IL-1β, IL-6, and TNF-α, and the chemokine MCP-1 (CCL-2) compared to those in cells stimulated with LPS only (Fig 4B-4E). The decrease in mRNA levels due to the extract was high, with values of approximately 90%, 97%, 80%, and 97% for IL-1 β, IL-6, TNF-α, and MCP-1, respectively.

**Fig 5:**
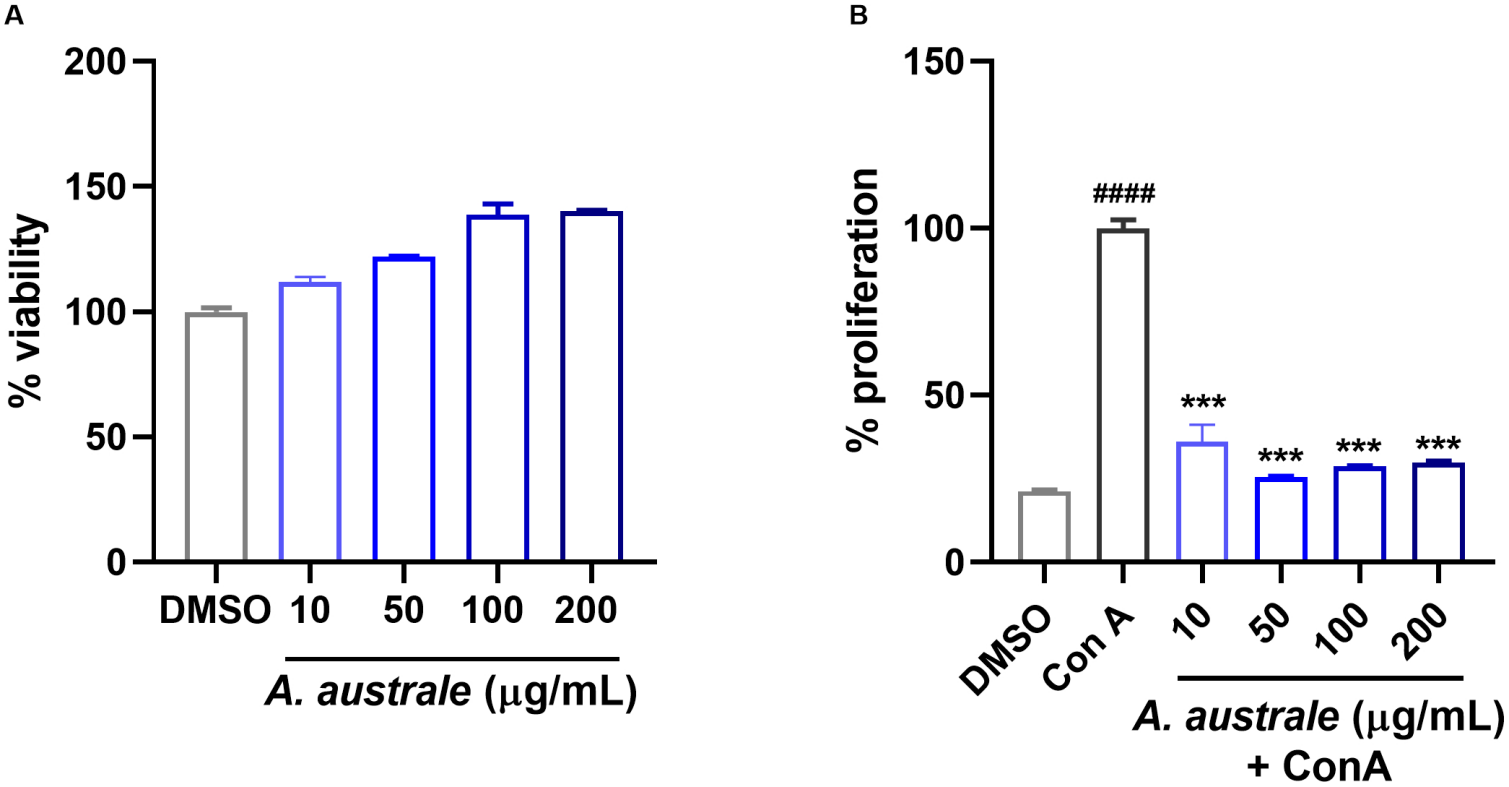
Effect of *A. australe* on T cell proliferation. **A)** Effect of *A. australe* extract on cell viability. The cells were treated with different concentrations of the extract. **(B)** The effect of *A. australe* on ConA-induced splenocyte proliferation. ANOVA (n = 3), #### P < 0.0001 vs. untreated cells. ***P < 0.001 vs. LPS-treated cells.

Further to the TLR signaling pathway, the T cell receptor signaling pathway was identified as a potential target of *A. australe*. Therefore, the effect of the extract on the regulation of T cell activation was examined. Lectin concanavalin A (ConA) binds to specific glycoproteins on the T cell surface, including the T cell receptor (TCR) complex, initiating various cellular responses such as proliferation [27]. For this reason, we used ConA-stimulated splenocytes, as an *in vitro* model of T cell activation and proliferation. No cytotoxic effects were observed at any of the tested concentrations (Fig 5A). When comparing the cells treated with *A. australe* to those treated with ConA alone, the extract significantly reduced the ConA-induced cell proliferation (Fig 5B).

The experimental findings corroborated the outcomes of the network pharmacology analysis, demonstrating that *A. australe* affects the TLR4 pathway and lymphocyte activation and proliferation. Overall, the extract was found to exhibit anti-inflammatory properties throughout various stages of the inflammatory process.

## Discussion

This study employed network pharmacology methods, UPLC-ESI-MS/MS analysis, and *in vitro* assays to explore the effects and mechanisms of *A. australe* in inflammation. Our findings indicate that pathways such as T-lymphocyte receptor activation and the TLR pathway are potential targets of *A. australe*, with *in vitro* assays confirming its effects on these targets.

*A. australe* is used in traditional medicine for various purposes, including its anti-inflammatory effects. For example, studies conducted in Paraguay on traditional markets selling medicinal plants identified the popular use of *A. australe* as an anti-inflammatory agent [9,10]. Recognizing the anti-inflammatory properties and targets of *A. australe* is crucial for validating its traditional use and identifying its potential therapeutic applications.

Using network pharmacology analysis, we found that the metabolites of *A. australe* target different pathways of the inflammatory process. Moreover, a highly complex network was detected, suggesting that *A. australe* acts at various levels. Clustering analysis revealed that cluster 3 was relevant to the inflammation process, highlighting the TLRs and TCR signaling pathways.

Firstly, TLR signaling plays a crucial role in the inflammatory process by orchestrating the production of pro-inflammatory cytokines and chemokines [28,29]. Specifically, monocytes, which are components of the innate immune system, are characterized by their substantial expression of TLRs, and they contribute to the inflammatory response by producing inflammatory mediators, including cytokines and chemokines [30]. These cells respond to various stimuli, including LPS, which is recognized by TLR4, leading to the activation of various signaling pathways. This activation, in turn, leads to the activation of nuclear factor-kappa B (NF-κB) and activator protein 1 (AP-1) and the release of pro-inflammatory mediators such as IL-1β, TNF-α, and MCP-1 [31]. In this study, the RT-qPCR results indicated that *A. australe* inhibited the expression of IL-1β, IL-6, TNF-α, and MCP-1 in LPS-treated monocyte cells. Consequently, we determined that the TLR pathway identified through network analysis was affected by *A. australe*, leading us to hypothesize that this species exerts anti-inflammatory effects by modulating the TLR signaling pathway.

The potential of *A. australe* extracts to inhibit the TLR4 pathway highlights that further investigation into its application as an anti-inflammatory agent is warranted. Indeed, several preclinical and clinical studies have demonstrated the potential of TLR antagonists and inhibitors for treating chronic inflammatory and autoimmune conditions [32]. For example, in rheumatic diseases, TLR inhibitors have shown promise in managing conditions such as rheumatoid arthritis and systemic lupus erythematosus [33]. However, there is currently a lack of effective TLR inhibitors for clinical use [34].

Secondly, the TCR pathway influences multiple aspects of T cell biology, including development, homeostasis, proliferation, differentiation, and effector functions. Dysregulation of TCR signaling can lead to the development of self-reactive T cells and contribute to the progression of autoimmune diseases [35]. In this work, *A. australe* was found to significantly decrease ConA-induced T cell proliferation. Importantly, the inhibition of the TCR signaling pathway has been shown to have potential for the treatment of autoimmune and inflammatory conditions. Normal quiescent lymphocytes undergo slow homeostatic proliferation to maintain constant T cell levels [36]. In contrast, activated lymphocytes proliferate rapidly in response to stimuli and are involved in signaling pathways other than homeostatic proliferation [37]. This difference explains the observed effects of the extract, which induced a slight increase in the number of cells on non-activated lymphocytes but induced a slight decrease in proliferation on activated lymphocytes. This latter scenario occurs in patients with inflammatory diseases, which cause lymphocytes to be activated and proliferate.

Overall, these results indicate that *A. australe* affects cells that are both part of the innate immune response (i.e., monocytes), and part of the adaptive response (i.e., lymphocytes). These findings are interesting because currently used anti-inflammatory drugs, such as corticosteroids, also have several cellular targets that support their anti-inflammatory action [38].

In the phytochemical analysis of the *A. australe* extract, 15 compounds were identified. Among these, caffeoylquinic acid derivatives, specifically 5-caffeoylquinic acid and two dicaffeoylquinic acids, were present. Caffeoylquinic acid and its derivatives have previously been documented to possess anti-inflammatory properties [39]. Notably, 4,5-dicaffeoylquinic acid has been shown to reduce the expression of inflammatory mediators, including nitric oxide synthase, cyclooxygenase-2, TNF-α, IL-1β, and IL-6 in murine RAW264.7 cells [40]. Additionally, caffeoylquinic acid derivatives have demonstrated efficacy in inhibiting carrageenan-induced edema and reducing the production of the pro-inflammatory cytokines TNF-α and IL-1β in an *in vivo* rat model [41]. In this study, we observed a decrease in TNF-α, IL-1β, and IL-6 levels in human monocytes treated with the *A. australe* extract. As such, these compounds may be responsible for the observed effects of the extract on cytokines. Caffeoyl hexoside was also identified in the extract, and caffeoyl glucosides have been demonstrated to mitigate LPS-induced inflammation in endothelial cells [42]. Therefore, these compounds may also contribute to the observed anti-inflammatory effects of the extract.

Additionally, this work found that the plant extract contained ferulic acid and kaempferol hexoside and both ferulic acid and kaempferol, along with their glycoside derivatives, have demonstrated anti-inflammatory properties in both *in vitro* and *in vivo* studies [43–45]. Therefore, these compounds may also contribute to the anti-inflammatory effects of the extract. In particular, kaempferol has been reported to inhibit ConA-activated T cell proliferation [45], suggesting that this compound may play a role in the observed effects of *A. australe* extract at the level of the T cell pathway.

The anti-inflammatory activity of the extract may be attributed to these compounds. However, it is crucial to acknowledge that the anti-inflammatory effects could result from the synergistic actions of multiple compounds present within the extract.

A limitation of this study is the absence of an evaluation of other critical pathways identified in the network pharmacology analysis as potential targets of the anti-inflammatory activity of *A. australe*. For instance, the effect of *A. australe* on the PI3K-Akt pathway could be assessed, given the association of this pathway with TLR activation. Furthermore, future research should assess the anti-inflammatory effects of *A. australe* across a broader spectrum of models, including *in vivo* studies.

In conclusion, this study demonstrated the anti-inflammatory properties of *A. australe* for the first time and identified its potential cellular targets, thereby supporting its traditional use and highlighting its potential as a therapeutic agent for inflammatory conditions.

## Supporting information

S1 Table Cellular targets of *A. australe* compounds identified using the Swiss TargetPrediction server. (XLSX)

S2 Table Functional enrichment analysis. (XLSX)

S3 Table Identification of the main compounds in *A. australe* methanolic extract using UPLC-ESI-MS/MS and HPLC-DAD. (DOCX)

S4 Fig The spectral data (LC-MS and LC-PDA) of the main compounds tentatively identified in the *A. australe* extract. (PDF)

S5 Fig Extracted ion chromatograms (EICs) in negative ionization for the compounds detected in the *A. australe* extract. (PDF)
