# Supplementary material for: Anti-inflammatory activity of Acanthospermum australe: Insights from network pharmacology, chemical analysis, and in vitro assays
Source: PLoS One. 2025 Nov 26;20(11):e0337712. doi: 10.1371/journal.pone.0337712 (PMC12654944; doi:10.1371/journal.pone.0337712)
Supplement: S3 Table — (DOCX) [file pone.0337712.s003.docx]

| **Peak** | **Rt (min)** | **[M-H]^-^** | **UVmax** | **MS/MS** | **Tentative identification** |
| --- | --- | --- | --- | --- | --- |
| 1 | 1.01 | 341.13 | 325,295sh | 179.68(100) | Caffeoyl hexoside |
| 2 | 1.82-1.85 | 353.37 | 326,295sh | 191.43(100), 179.36(5) | 5-caffeoylquinic acid^a^ |
| 3 | 2.32 | 179.00 | 326, 295sh | 134.35(100) | Caffeic acid |
| 4 | 4.54 | 193.30 | 324,292sh | 192.63(10), 161.61(35), 133.34(100) | Ferulic acid |
| 5 | 4.79-4.86 | 515.48 | 328, 290sh | 353.43(100), 179.49(25) | Dicaffeoylquinic acid 1 |
| 6 | 5.12 | 609.59 | 353, 267, 255 | 609.63(10), 300.68(100) | Quercetin rutinoside |
| 7 | 5.13 | 463.37 | 353, 267, 255 | 301.59(100) | Quercetin hexoside |
| 8 | 5.33-5.41 | 515.48 | 330, 290sh | 515.80(10), 353.59(100) | Dicaffeoylquinic acid 2 |
| 9 | 5.80 | 447.54 |  | 447.29(100), 284.76(30) | Kaempferol hexoside |
| 10 | 6.69 | 345.39 |  | 330.55(100), 315.42(20), 286.91(5) | 5, 7, 3’, 4’-tetrahydroxy-3,6-dimethoxy flavone (axillarin) |
| 11 | 7.28 | 329.30 | 340,269 | 314.12(40), 299.45(95), 271.54(100) | 5, 7, 4’-trihydroxy-3,6-dimethoxy flavone |
| 12 | 7.59 | 663.84 |  | 618.27(100) | Unknown |
| 13 | 7.59 | 343.00 |  |  | Penduletin |
| 14 | 8.60 | 663.84 |  | 617.94(100), 241.91(15) | Unknown |
| 15 | 9.28 | 293.54 |  | 127.32(100) | Unknown |

**S3 Table. Tentative identification of the main compounds in *Acanthospermum australe* methanolic extract using UPLC-ESI-MS/MS and HPLC-DAD.**

^a^According to Clifford et al. (2003)
