# Supplementary material for: Anti-inflammatory activity of Acanthospermum australe: Insights from network pharmacology, chemical analysis, and in vitro assays
Source: PLoS One. 2025 Nov 26;20(11):e0337712. doi: 10.1371/journal.pone.0337712 (PMC12654944; doi:10.1371/journal.pone.0337712)
Supplement: S4 Fig — (PDF) [file pone.0337712.s004.pdf]

S4 Figure. The spectral data (LC-MS and LC-PDA) of the main compounds tentatively identified in the *A. australe* extract.

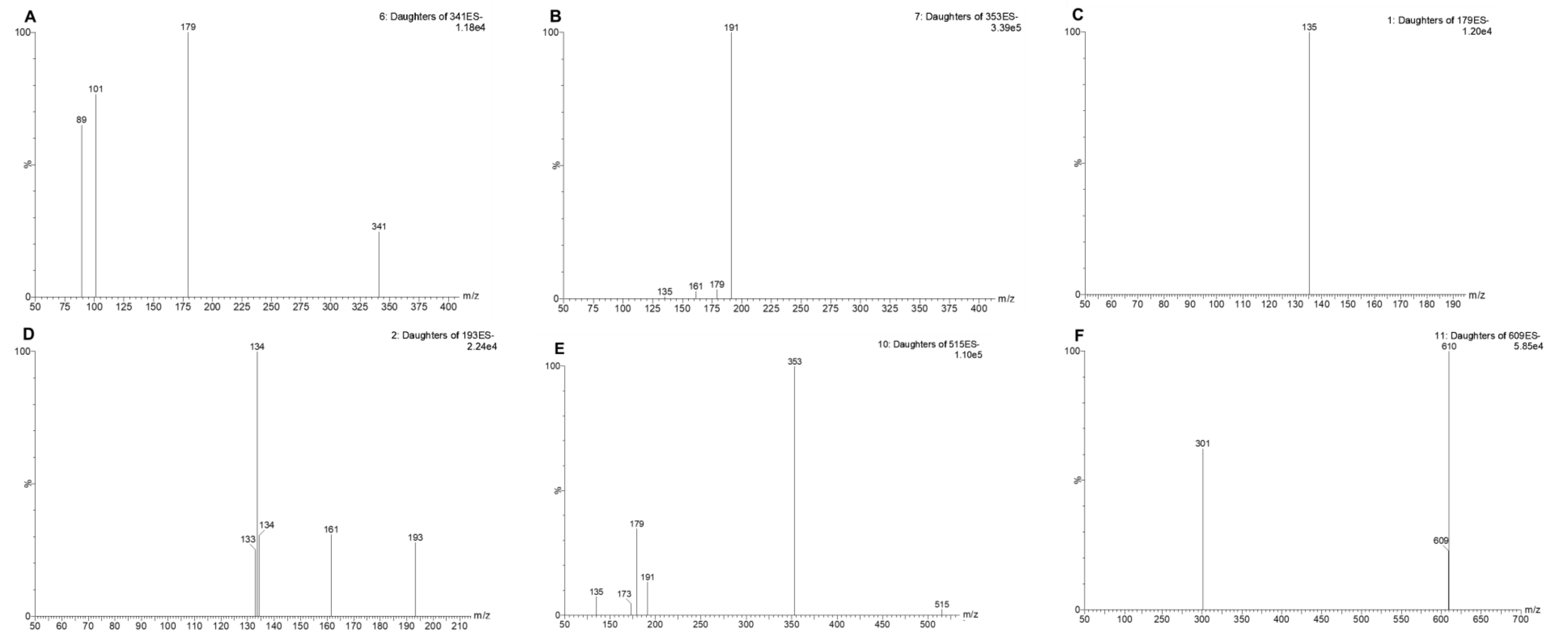

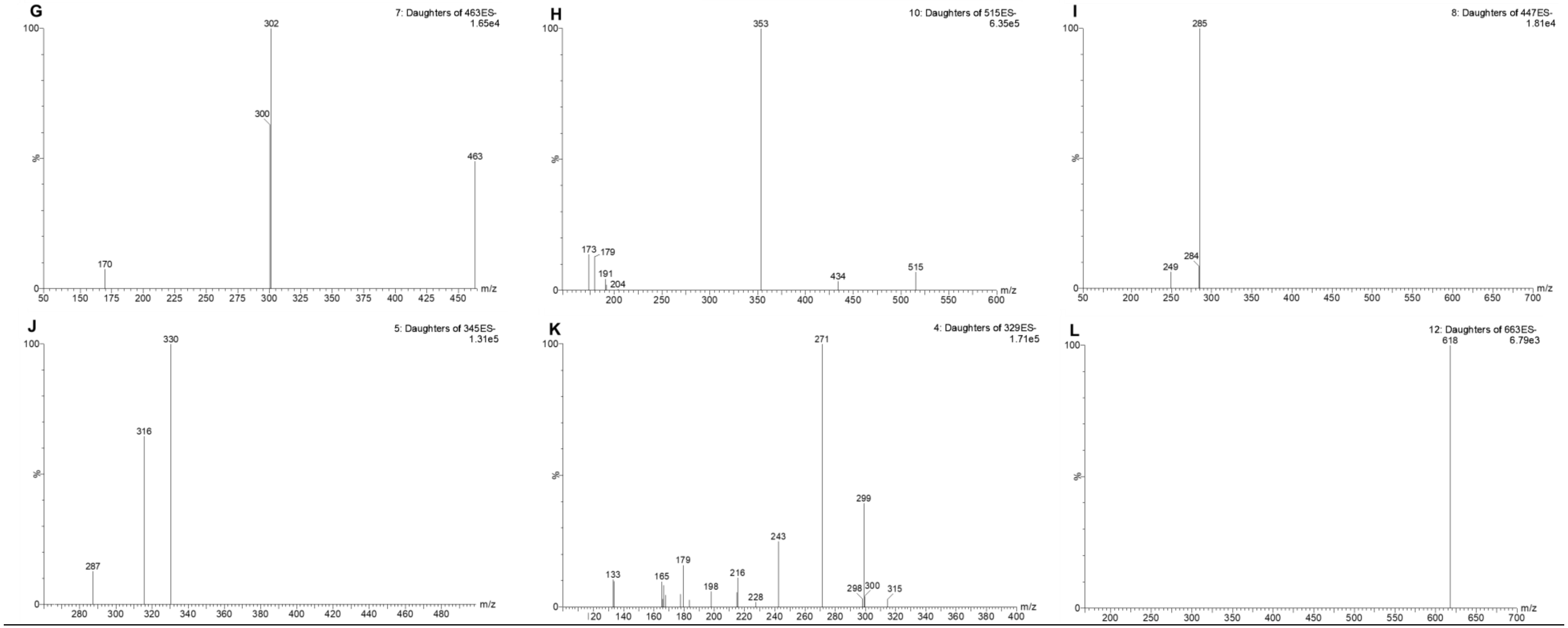

G
7: Daughters of 463ES-
1.65e4
302
300
463
170
%
m/z
H
10: Daughters of 515ES-
6.35e5
353
173
179
191
204
434
515
%
m/z
I
8: Daughters of 447ES-
1.81e4
285
249
284
%
m/z
J
5: Daughters of 345ES-
1.31e5
330
316
287
%
m/z
K
4: Daughters of 329ES-
1.71e5
271
299
243
133
165
179
198
216
228
298
300
315
%
m/z
L
12: Daughters of 663ES-
6.79e3
618
%
m/z

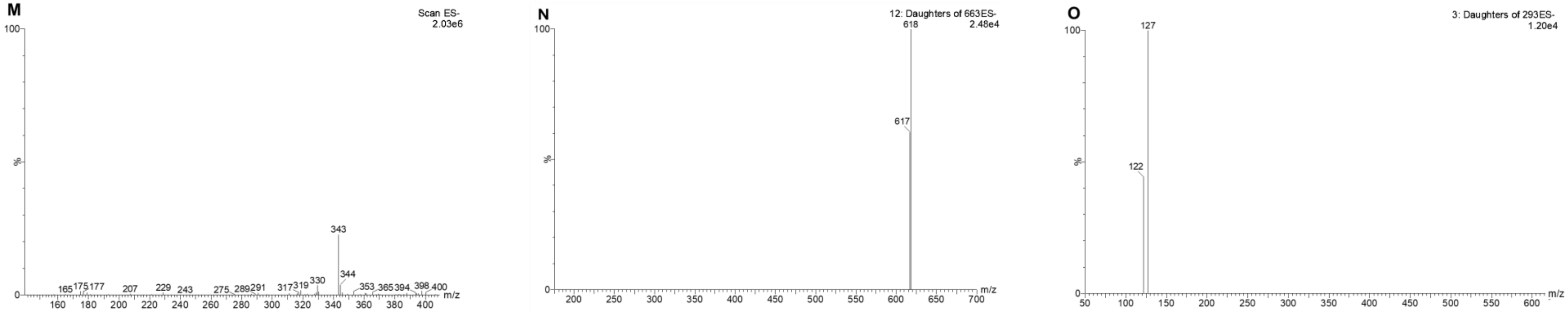
M
Scan ES-
2.03e6
343
344
330
165 175 177 207 229 243 275 289 291 317 319 353 365 394 398 400
m/z
N
12: Daughters of 663ES-
2.48e4
618
617
m/z
O
3: Daughters of 293ES-
1.20e4
127
122
m/z
%

UV spectra

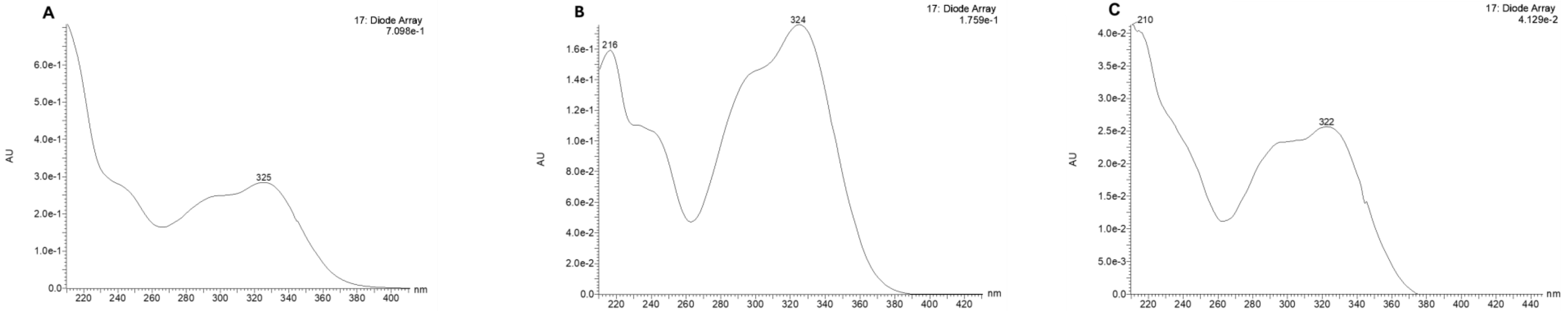
A
17: Diode Array
7.098e-1
325
B
17: Diode Array
1.759e-1
324
216
C
17: Diode Array
4.129e-2
210
322
AU
nm

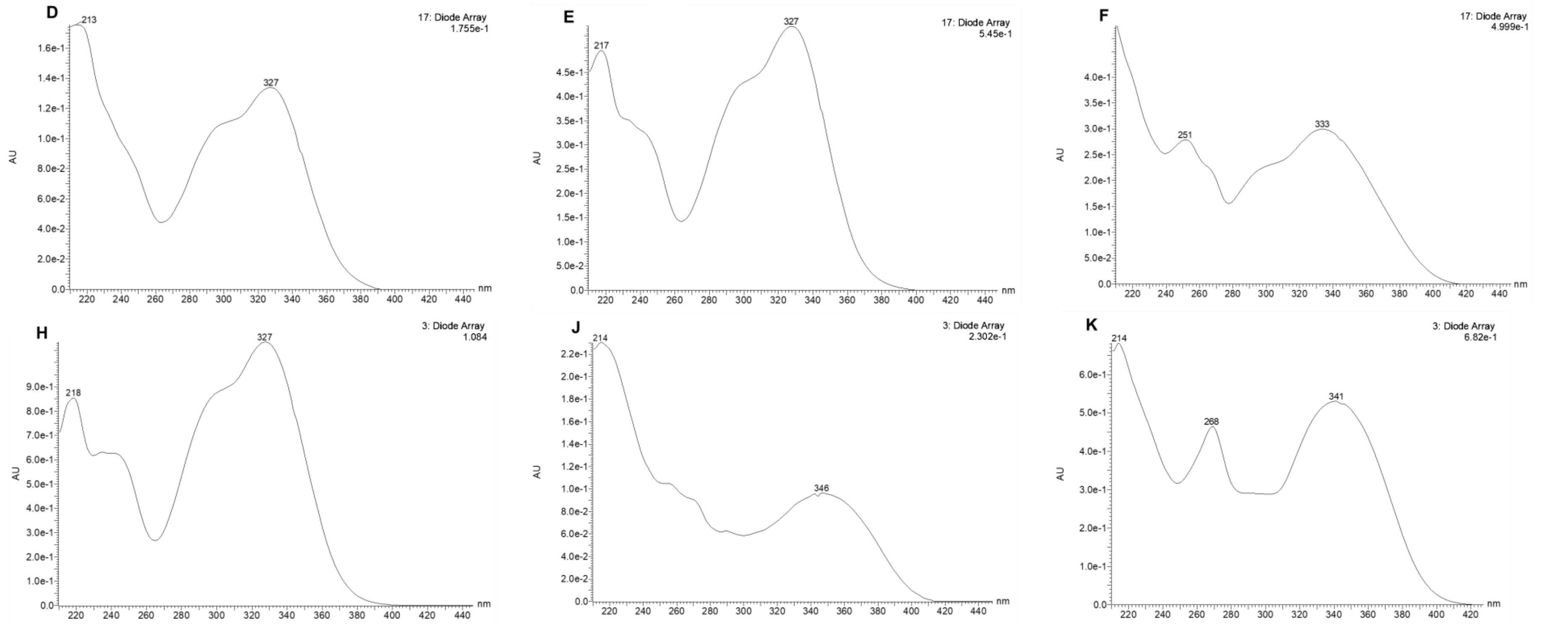

D
213
327
17: Diode Array
1.755e-1
AU
nm
E
217
327
17: Diode Array
5.45e-1
AU
nm
F
251
333
17: Diode Array
4.999e-1
AU
nm
H
218
327
3: Diode Array
1.084
AU
nm
J
214
346
3: Diode Array
2.302e-1
AU
nm
K
214
268
341
3: Diode Array
6.82e-1
AU
nm

M

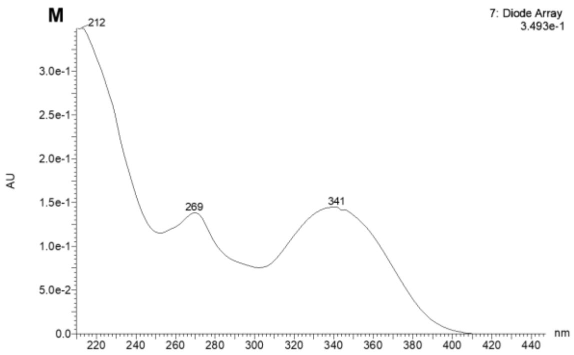

***Abbreviations: A:** Caffeoyl hexoside; **B:** 5-caffeoylquinic acid; **C:** caffeic acid; **D:** ferulic acid; **E:** Dicaffeoylquinic acid 1; **F:** Quercetin rutinoside; **G:** Quercetin hexoside; **H:** Dicaffeoylquinic acid 2; **I:** kaempferol hexoside; **J:** axillarin; **K:** 5, 7, 4’-trihydroxy-3,6-dimethoxy flavone; **L:** Unknown 1; **M:** Penduletin; **N:** Unknown 2; **O:** Unknown 3.
