# Supplementary material for: Anti-inflammatory activity of Acanthospermum australe: Insights from network pharmacology, chemical analysis, and in vitro assays
Source: PLoS One. 2025 Nov 26;20(11):e0337712. doi: 10.1371/journal.pone.0337712 (PMC12654944; doi:10.1371/journal.pone.0337712)
Supplement: S5 Fig — (PDF) [file pone.0337712.s005.pdf]

S5 Figure. Extracted ion chromatograms (EICs) in negative ionization for the compounds detected in the *A. australe* extract.

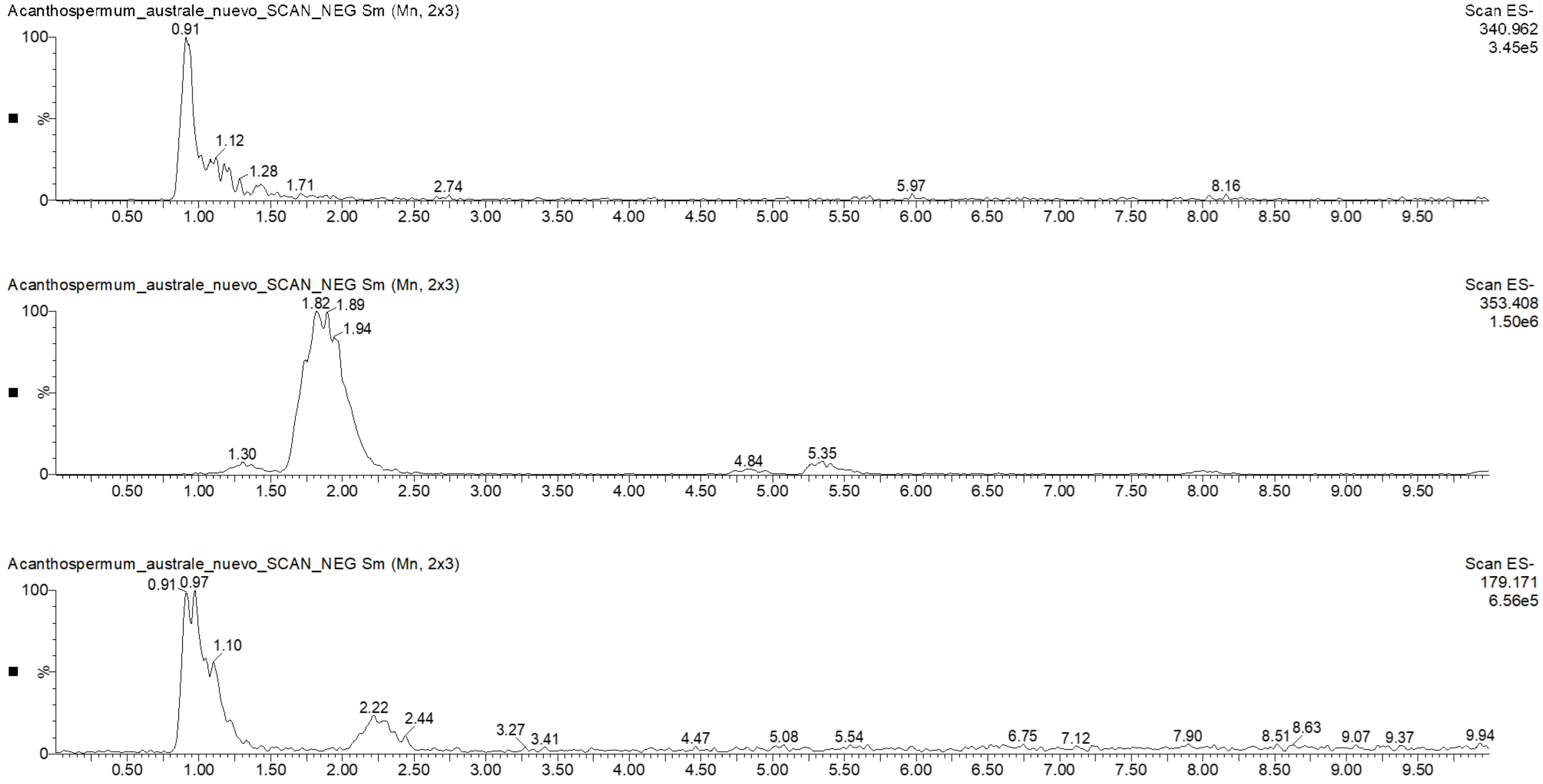

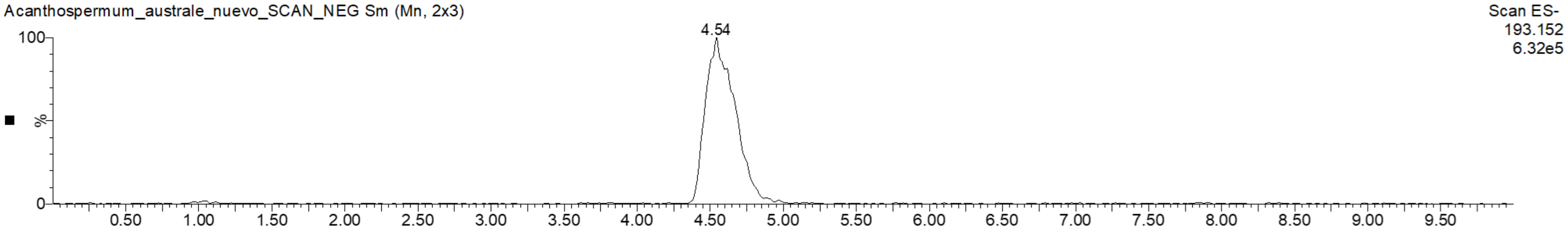
Acanthospermum_australe_nuevo_SCAN_NEG Sm (Mn, 2x3)
Scan ES-
193.152
6.32e5
4.54
100
%
0
0.50 1.00 1.50 2.00 2.50 3.00 3.50 4.00 4.50 5.00 5.50 6.00 6.50 7.00 7.50 8.00 8.50 9.00 9.50

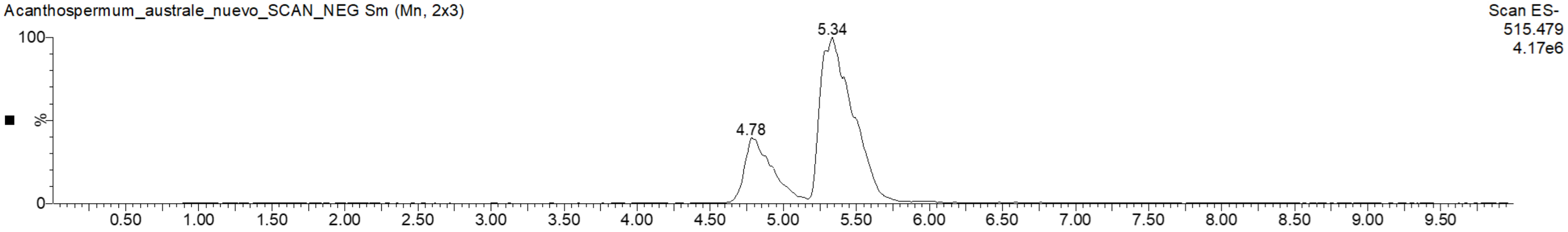
Acanthospermum_australe_nuevo_SCAN_NEG Sm (Mn, 2x3)
Scan ES-
515.479
4.17e6
5.34
4.78
100
%
0
0.50 1.00 1.50 2.00 2.50 3.00 3.50 4.00 4.50 5.00 5.50 6.00 6.50 7.00 7.50 8.00 8.50 9.00 9.50

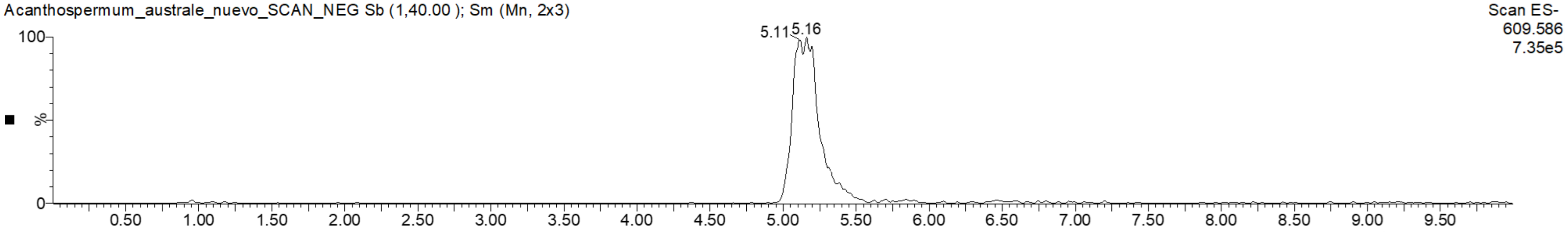
Acanthospermum_australe_nuevo_SCAN_NEG Sb (1,40.00); Sm (Mn, 2x3)
Scan ES-
609.586
7.35e5
5.11
5.16
100
%
0
0.50 1.00 1.50 2.00 2.50 3.00 3.50 4.00 4.50 5.00 5.50 6.00 6.50 7.00 7.50 8.00 8.50 9.00 9.50

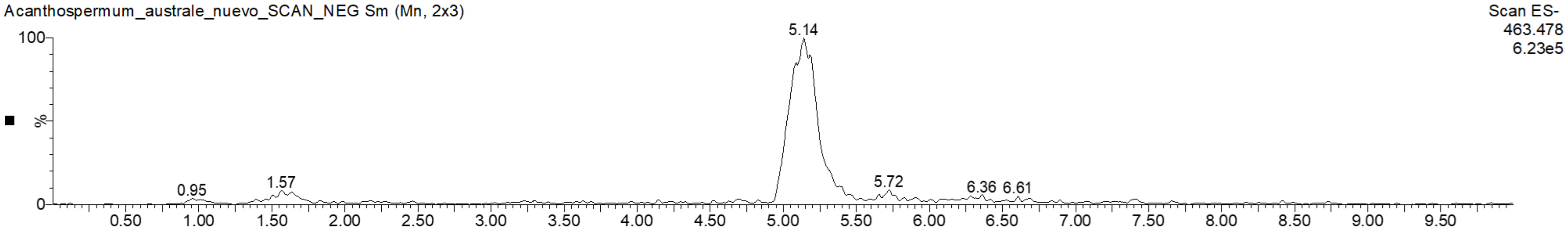
Acanthospermum_australe_nuevo_SCAN_NEG Sm (Mn, 2x3)
Scan ES-
463.478
6.23e5
100
%
0
5.14
0.95
1.57
5.72
6.36
6.61
0.50 1.00 1.50 2.00 2.50 3.00 3.50 4.00 4.50 5.00 5.50 6.00 6.50 7.00 7.50 8.00 8.50 9.00 9.50

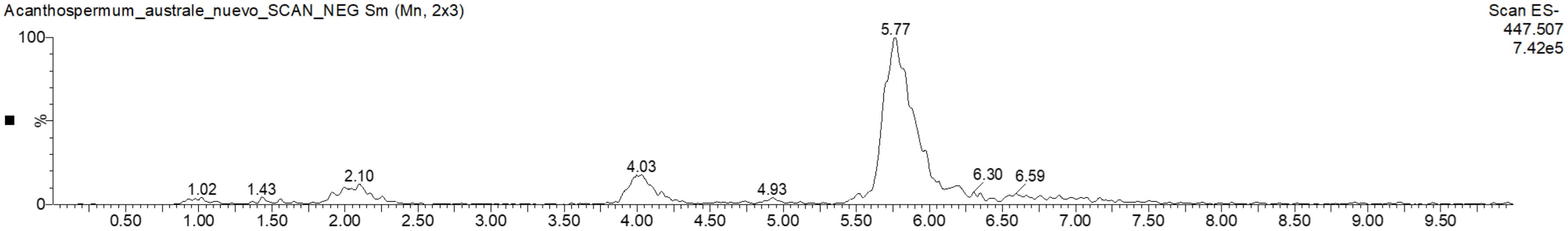
Acanthospermum_australe_nuevo_SCAN_NEG Sm (Mn, 2x3)
Scan ES-
447.507
7.42e5
100
%
0
5.77
1.02
1.43
2.10
4.03
4.93
6.30
6.59
0.50 1.00 1.50 2.00 2.50 3.00 3.50 4.00 4.50 5.00 5.50 6.00 6.50 7.00 7.50 8.00 8.50 9.00 9.50

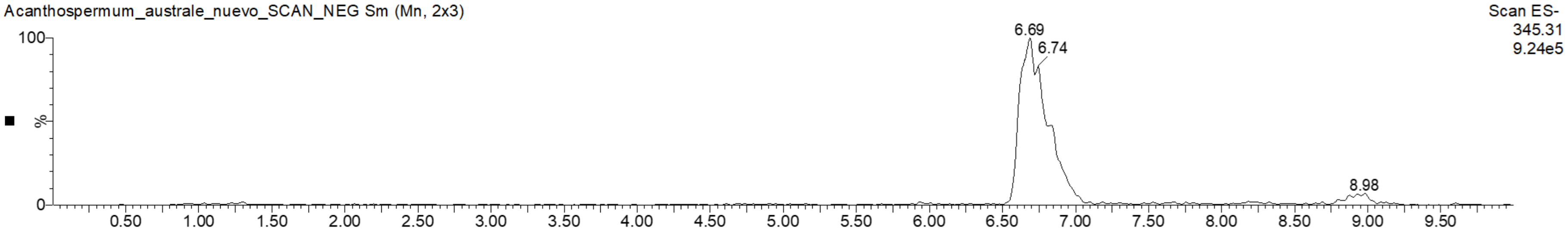
Acanthospermum_australe_nuevo_SCAN_NEG Sm (Mn, 2x3)
Scan ES-
345.31
9.24e5
100
%
0
6.69
6.74
8.98
0.50 1.00 1.50 2.00 2.50 3.00 3.50 4.00 4.50 5.00 5.50 6.00 6.50 7.00 7.50 8.00 8.50 9.00 9.50

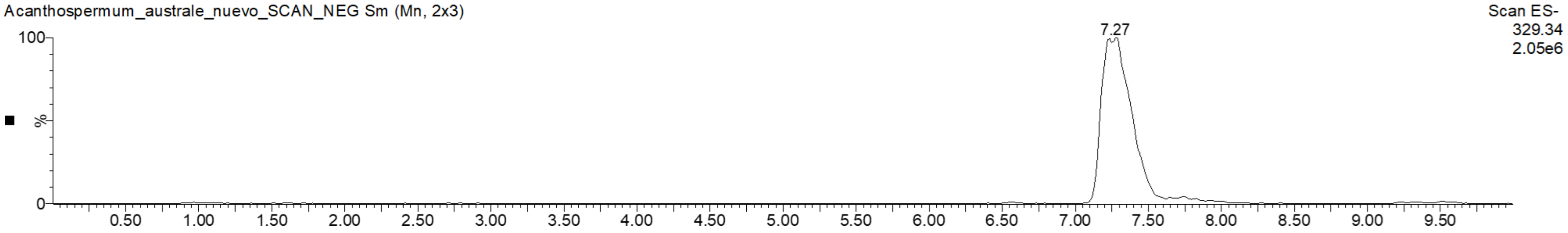
Acanthospermum_australe_nuevo_SCAN_NEG Sm (Mn, 2x3)
Scan ES-
329.34
2.05e6
100
%
0
7.27
0.50
1.00
1.50
2.00
2.50
3.00
3.50
4.00
4.50
5.00
5.50
6.00
6.50
7.00
7.50
8.00
8.50
9.00
9.50

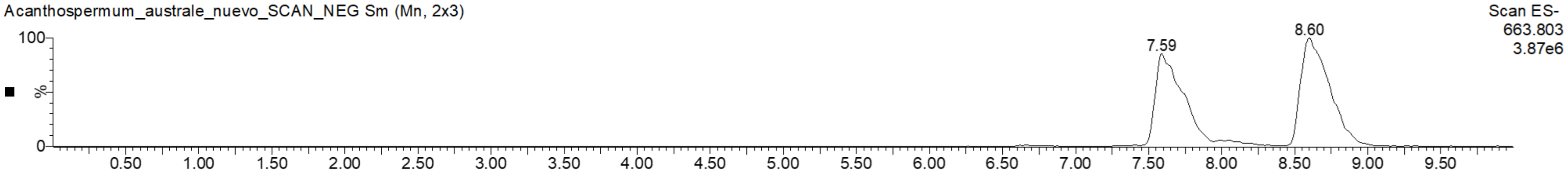
Acanthospermum_australe_nuevo_SCAN_NEG Sm (Mn, 2x3)
Scan ES-
663.803
3.87e6
100
%
0
7.59
8.60
0.50
1.00
1.50
2.00
2.50
3.00
3.50
4.00
4.50
5.00
5.50
6.00
6.50
7.00
7.50
8.00
8.50
9.00
9.50

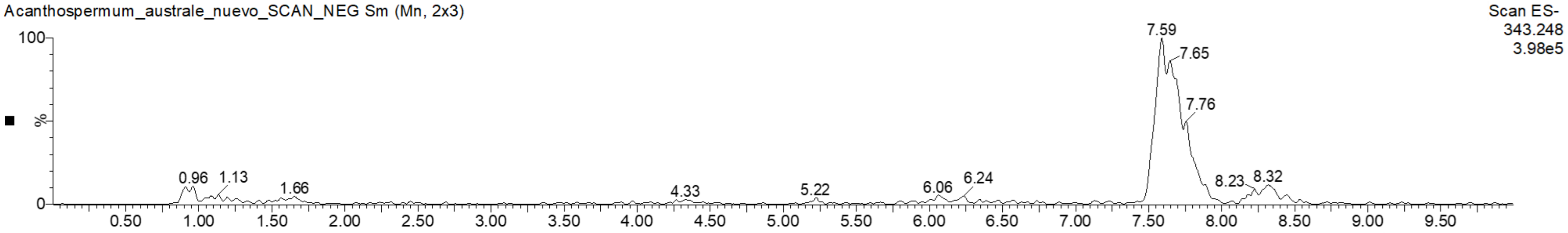
Acanthospermum_australe_nuevo_SCAN_NEG Sm (Mn, 2x3)
Scan ES-
343.248
3.98e5
100
%
0
0.96
1.13
1.66
4.33
5.22
6.06
6.24
7.59
7.65
7.76
8.23
8.32
0.50
1.00
1.50
2.00
2.50
3.00
3.50
4.00
4.50
5.00
5.50
6.00
6.50
7.00
7.50
8.00
8.50
9.00
9.50

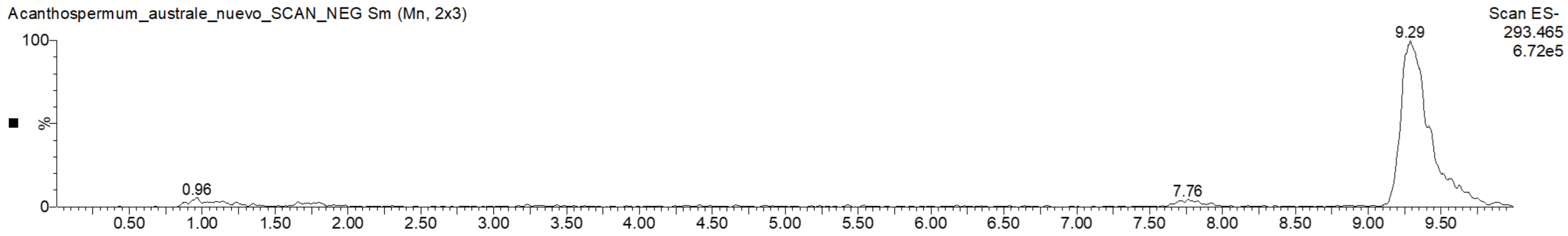
Acanthospermum_australe_nuevo_SCAN_NEG Sm (Mn, 2x3)
Scan ES-
293.465
6.72e5
100
%
0
9.29
0.96
7.76
0.50
1.00
1.50
2.00
2.50
3.00
3.50
4.00
4.50
5.00
5.50
6.00
6.50
7.00
7.50
8.00
8.50
9.00
9.50
